# Time to Reperfusion Dictates Cardiac Function and Myocardial Strain in a 7-Tesla Magnetic Resonance Imaging Rat Model

**DOI:** 10.3390/jcdd13010010

**Published:** 2025-12-22

**Authors:** Mako Ito, Junpei Ueda, Sei Yasuda, Isamu Yabata, Koji Itagaki, Natsuo Banura, Shigeyoshi Saito

**Affiliations:** 1 Department of Medical Physics and Engineering, Division of Health Sciences, The University of Osaka Graduate School of Medicine, Osaka 565-0871, Japan; u963814d@ecs.osaka-u.ac.jp (M.I.); uedaj@sahs.med.osaka-u.ac.jp (J.U.); u305649g@ecs.osaka-u.ac.jp (S.Y.); i_yabata@hp-rad.med.osaka-u.ac.jp (I.Y.); beitou@kuhp.kyoto-u.ac.jp (K.I.); banura.natsuo@ncvc.go.jp (N.B.); 2 Department of Radiological Sciences, Faculty of Health Sciences, Morinomiya University of Medical Sciences, Osaka 559-8611, Japan; 3 Department of Radiology, The University of Osaka Hospital, Osaka 565-0871, Japan; 4 Division of Clinical Radiology Service, Kyoto University Hospital, Kyoto 606-8507, Japan; 5 Department of Advanced Medical Technologies, National Cerebral and Cardiovascular Research Center, Osaka 564-8565, Japan; 6 Immunology Frontier Research Center, The University of Osaka, 3-1 Yamadaoka, Osaka 565-0871, Japan; 7 World Premier International Research Center Initiative Premium Research Institute for Human Metaverse Medicine, The University of Osaka, 2-2 Yamadaoka, Osaka 565-0871, Japan

**Keywords:** coronary artery reperfusion, Wistar rats, 7-tesla magnetic resonance imaging, strain, myocardial infarction, fibrosis

## Abstract

This study used a rat model of coronary artery reperfusion imaged with preclinical 7-tesla magnetic resonance imaging (7T-MRI) to evaluate cardiac function, myocardial deformation, and the impact of infarction-to-reperfusion time. Wistar rats were assigned to control (*n* = 6), 20 min infarction (*n* = 10), 30 min infarction (*n* = 6), and 40 min infarction (*n* = 6) groups. Myocardial infarction occurred in all infarction groups but not in controls. Imaging included short- and long-axis slices. Cardiac function was assessed using end-diastolic volume, end-systolic volume, and left-ventricular ejection fraction. Myocardial deformation was analyzed by circumferential strain, radial strain (RS), and longitudinal strain (LS, four-chamber and two-chamber) using feature tracking. The 30 and 40 min infarction groups showed significant reductions in cardiac function and strain compared to the controls. RS decreased significantly between the control and 20 min infarction groups (40.6 ± 4.7% and 34.0 ± 4.1%, *p* < 0.05). No significant LS difference was observed between 30 and 40 min. Consequently, RS detects early myocardial changes (20 min), whereas LS may reflect compensatory contractility in severe infarction. Preclinical 7T-MRI provides valuable insights into the impact of infarction duration on cardiac function and myocardial deformation.

## 1. Introduction

According to the Ministry of Health, Labor, and Welfare’s “Summary of Vital Statistics” for fiscal year 2021, approximately 210,000 individuals die annually in Japan from heart disease. Approximately 14% of these deaths are attributable to acute myocardial infarction, accounting for approximately 30,000 fatalities. Myocardial infarction occurs when a coronary artery is occluded, preventing blood flow to the supplied myocardium, resulting in myocardial necrosis and impaired cardiac function. To salvage ischemic myocardium, it is essential to restore blood flow through coronary revascularization [1]. The “time to reperfusion” significantly influences severity [2,3]. Infarct size reflects the combined effects of ischemia- and reperfusion-induced injury [4]. Ischemic injury depends on the duration of ischemia and residual blood flow, whereas reperfusion injury depends on the duration and severity of preceding ischemia. The greater the ischemic injury, the lower the myocardial salvage rate. Although early reperfusion is crucial, reperfusion itself may also cause injury.

Echocardiography, computed tomography (CT), and cardiac magnetic resonance imaging (MRI) are used to evaluate the ischemic myocardium. Cardiac MRI is widely applied in clinical practice to assess cardiac function via left-ventricular ejection fraction (EF) and to evaluate myocardial properties using strain analysis and late gadolinium-enhanced MRI [5]. Cardiac function is typically assessed by end-diastolic volume (EDV), end-systolic volume (ESV), and EF to evaluate ventricular systolic function [6]. Strain is measured along three axes: circumferential strain (CS), radial strain (RS), and longitudinal strain (LS), allowing quantitative evaluation of regional wall motion [7,8]. Although echocardiography provides similar information, results vary considerably with operator skill and equipment settings. In contrast, cardiac MRI offers high reproducibility and accuracy [9], and unlike CT, does not involve radiation exposure.

Validating the relationship between reperfusion time and severity in humans is challenging, owing to variability in disease onset. Therefore, coronary artery reperfusion models are used in animal experiments [10]. In this model, the left main coronary artery is occluded for a defined period and then reperfused to simulate MI and subsequent reperfusion. A previous study using echocardiography and MRI demonstrated a reduction in EF in a rat model subjected to 30 min of infarction [11]. However, previous studies were limited by fixed reperfusion times and focused primarily on cardiac function without evaluating myocardial deformation. This study aimed to assess cardiac function in a coronary artery reperfusion rat model using preclinical 7-tesla MRI (7T-MRI) (PharmaScan 70/16 US; Bruker Biospin, Ettlingen, Germany) and to examine the relationship between infarction-to-reperfusion time and cardiac outcomes, considering both cardiac function and myocardial deformation. This study’s incremental contribution is to systematically relate graded ischemia durations to both function and multi-axis strain at a fixed subacute time, revealing RS as an earlier discriminator than GLS/CS/LVEF and aligning imaging readouts with histology.

## 2. Materials and Methods

### 2.1. Animal Preparation

This study was conducted in accordance with the Animal Research: Reporting of In Vivo Experiments guidelines. All experimental protocols were approved by the Research Ethics Committee of Osaka University, Osaka, Japan (Approval Number: R02-05-0). Animal procedures were performed in accordance with the Osaka University Guidelines for Animal Experimentation and the National Institutes of Health Guide for the Care and Use of Laboratory Animals. Male Wistar rats (8–11 weeks old, 195–242 g; Japan SLC, Hamamatsu, Japan) were housed in a controlled environment (24 °C; 12:12 h light/dark cycle) with free access to standard pellets and water. Rats were divided into control (*n* = 6) and myocardial infarction–reperfusion groups: 20 min (*n* = 10), 30 min (*n* = 6), and 40 min (*n* = 6). For model preparation, a left thoracotomy was performed to expose the ribs, with an incision made in the intercostal space between the fourth and fifth ribs. The chest was then opened to reveal the heart, and the left coronary artery was ligated with suture material for a specified duration to create an ischemia–reperfusion injury model. The models were ligated for 20, 30, or 40 min. During surgery and imaging, animals were maintained on isoflurane (3.0% induction; 1.5–2.0% maintenance), body temperature was servo-controlled (36.5 °C), and respiratory motion was continuously monitored. Analgesia (buprenorphine, 0.05 mg/kg) was administered preoperatively and postoperatively. Successful ischemia was confirmed by immediate regional pallor and wall motion reduction on intraoperative visual inspection, followed by cine-MRI-detected hypokinesia. The chest was subsequently closed, and the skin wound was sutured. MRI was performed 1 week postoperatively. Hearts from both control animals and coronary artery reperfusion models subjected to 20, 30, and 40 min of infarction at the level of the cardiac papillary muscle were excised and stained with Sirius Red and hematoxylin and eosin (HE) following the MRI study. Rats were randomly allocated to control, 20, 30, or 40 min occlusion groups using a random-number generator. MRI data analysis and histological evaluation were performed by investigators blinded to group assignment. MRI was performed at 1 week to target the early subacute phase after ischemia–reperfusion, thereby reducing confounding from immediate post-operative physiology and facilitating standardized strain analysis. A follow-up study is planned at 24–48 h to characterize acute-phase strain behavior.

### 2.2. MRI

Magnetic resonance (MR) images of animal hearts were acquired using a horizontal 7-T scanner (PharmaScan 70/16 US; Bruker Biospin, Ettlingen, Germany) equipped with a 60 mm inner-diameter volume coil. Rats were positioned in a stereotaxic frame with a mouthpiece to minimize motion during image acquisition [12,13,14]. Body temperature was maintained at 36.5 °C with regulated water flow and continuously monitored using a physiological monitoring system (SA Instruments Inc., Stony Brook, NY, USA). All cardiac MR experiments were performed under general anesthesia induced with isoflurane (Viatris Inc., Tokyo, Japan, 3.0% for induction and 2.0% for maintenance). After MRI, rats were euthanized under deep anesthesia with 5% isoflurane.

Short-axis images were obtained using fast low-angle shot (FLASH) with navigator echo (IntraGate, Bruker, Bruker Biospin, Ettlingen, Germany) under the following parameters: repetition time (TR) = 44 ms, echo time (TE) = 2.2 ms, flip angle = 25°, 14 movie frames per cardiac cycle, field of view (FOV) = 42 × 42 mm^2^, resolution = 219 µm × 219 µm, matrix = 192 × 192, five slices spanning the heart from apex to base, oversampling = 250, and total scan time = 21 min 20 s.

Long-axis views (four-chamber and two-chamber) were obtained using FLASH with navigator echo (IntraGate, Bruker) under the following parameters: TR = 6.5 ms, TE = 3.0 ms, flip angle = 10°, 14 movie frames per cardiac cycle, FOV = 42 × 42 mm^2^, resolution = 219 µm × 219 µm, matrix = 192 × 192, one slice, oversampling = 250, and scan time = 3 min 37 s. The total scan time per animal was approximately 30 min.

Image analysis was performed using CVi42 (Circle Cardiovascular Imaging Inc., Calgary, AB, USA). In each slice of the left-ventricular short-axis image, the left-ventricular cavity and epicardium were manually contoured at end-systole and end-diastole, and ESV, EDV, and EF were calculated to evaluate cardiac function by two observers. CS and RS were also calculated using the feature-tracking method to assess myocardial deformation. In the four-chamber and two-chamber long-axis views, the left-ventricular cavity and epicardial borders were manually contoured at end-systole and end-diastole, and ESV, EDV, and EF were calculated.

### 2.3. Statistical Analysis

Data are presented as mean ± standard deviation. Differences among groups were compared using one-way analysis of variance followed by Tukey’s post hoc test for ESV, EDV, EF, CS, RS, four-chamber LS, and two-chamber LS. All analyses were performed using Prism 9 software (version 9, GraphPad Software, CA, USA). Statistical significance was set at *p* < 0.05.

A total of 28 rats were enrolled, and no animals died during reperfusion or were excluded from the study due to poor image quality. All 28 animals (control = 6, 20 min = 10, 30 min = 6, 40 min = 6) were included in the final analysis. Sample-size adequacy was evaluated post hoc (G*Power 3.1, Heinrich-Heine-Universität Düsseldorf, Düsseldorf, Germany); the achieved power for radial strain differences between control and 20 min groups was 0.86 (effect size 1.45, α = 0.05).

## 3. Results

### 3.1. Number of Animals and Body Weights

The average body weight of the control group was 216.7 ± 20.2 g, whereas those of the 20, 30, and 40 min infarction groups were 223.2 ± 10.6 g, 229.2 ± 8.3 g, and 228.2 ± 5.7 g, respectively.

### 3.2. Cardiac Function

**Figure 1:**
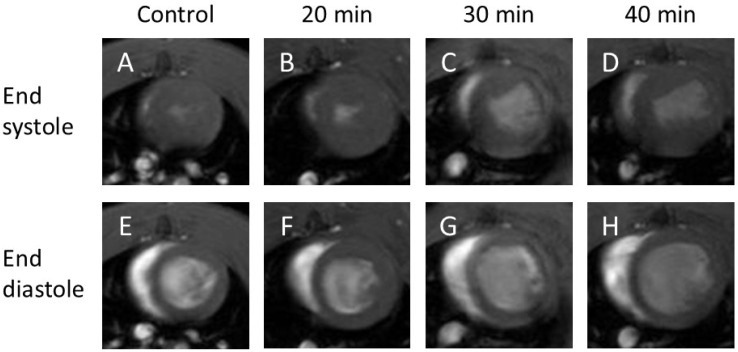
Cine-MR images. Cine images of end-systole (**A**–**D**) and end-diastole (**E**–**H**). At end-systole, infarction at 30 min (**C**) and 40 min (**D**) showed no contraction compared with the control (**A**) and 20 min infarction (**B**). At end-diastole, infarction at 30 min (**G**) and 40 min (**H**) showed increased volume compared with the control (**E**) and 20 min infarction (**F**).

End-systolic cine images (Figure 1A–D) showed no decrease in cardiac function at 20 min after infarction (Figure 1B), but a clear reduction at 30 min (Figure 1C) and 40 min (Figure 1D) compared with the control (Figure 1A).

**Figure 2:**
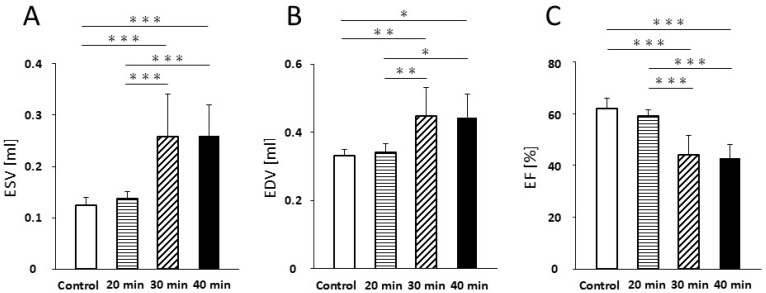
Cardiac function. ESV (**A**) increased at 30 min and 40 min of infarction compared with the control and 20 min infarction (*p* < 0.001). EDV (**B**) increased at 30 min (*p* < 0.01) and 40 min (*p* < 0.05) of infarction compared with the control and 20 min infarction. EF (**C**) decreased at 30 min and 40 min of infarction compared with the control and 20 min infarction (*p* < 0.001). Abbreviations: ESV, end-systolic volume; EDV, end-diastolic volume; EF, ejection fraction * *p* < 0.05; ** *p* < 0.01; *** *p* < 0.001.

Regarding ESV, no significant difference was detected between the control and 20 min infarction groups (0.13 ± 0.02 mL vs. 0.14 ± 0.01 mL; Figure 2A). However, ESV was significantly increased in the 30 min group (0.26 ± 0.08 mL; *p* < 0.001) and 40 min group (0.26 ± 0.06 mL; *p* < 0.001) compared with both the control and 20 min groups. No significant difference was found between the 30 and 40 min groups (Figure 2A).

End-diastolic cine images (Figure 1E–H) revealed no visual changes between the control (Figure 1E) and 20 min infarction (Figure 1F) groups; however, an increased volume was observed in the 30 min (Figure 1G) and 40 min (Figure 1H) groups compared to both the control and 20 min groups.

Similarly, no significant difference in EDV was detected between the control and 20 min groups (0.33 ± 0.02 mL vs. 0.34 ± 0.02 mL; Figure 2B, Table 1). A significant increase in EDV was noted in the 30 min group (0.45 ± 0.08 mL; *p* < 0.01) and the 40 min group (0.44 ± 0.07 mL; *p* < 0.05) compared to the control and 20 min groups, with no significant difference between the 30 and 40 min groups (Figure 2B, Table 1).

For EF, no significant difference was found between the control and 20 min groups (62.1 ± 3.8% vs. 59.2 ± 2.2%; Figure 2C, Table 1). In contrast, EF was significantly reduced in the 30 min group (44.0 ± 7.8%, *p* < 0.001) and 40 min group (42.7 ± 5.4%; *p* < 0.001) compared to both the control and 20 min groups. No significant difference was observed between the 30 and 40 min groups (Figure 2C, Table 1).

### 3.3. Myocardial Strain

**Figure 3:**
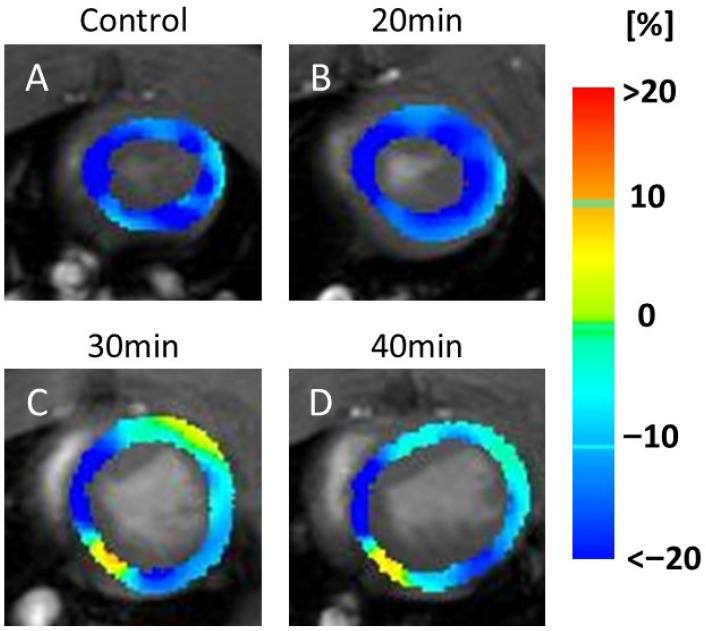
Color maps of circumferential strain (CS). The image shows a series of MRI scans of the heart, labeled (**A**–**D**), representing the control and 20, 30, and 40 min post-infarction groups, respectively. Each scan is overlaid with a color map indicating strain values in the heart muscle: blue (<−20%), green (0%), and red (>20%). The scans illustrate changes in strain distribution over time, which are relevant for assessing cardiac function. Yellow indicates motion decline, which was observed at 30 min (**C**) and 40 min (**D**) after infarction compared to the control (**A**) and 20 min (**B**). Abbreviation: MRI, magnetic resonance imaging.

In the CS maps, no visual changes were detected between the controls (Figure 3A) and 20 min infarction groups (Figure 3B). However, visible reductions in the yellow areas (indicating lower CS values) were observed in the 30 min (Figure 3C) and 40 min (Figure 3D) infarction groups.

**Figure 4:**
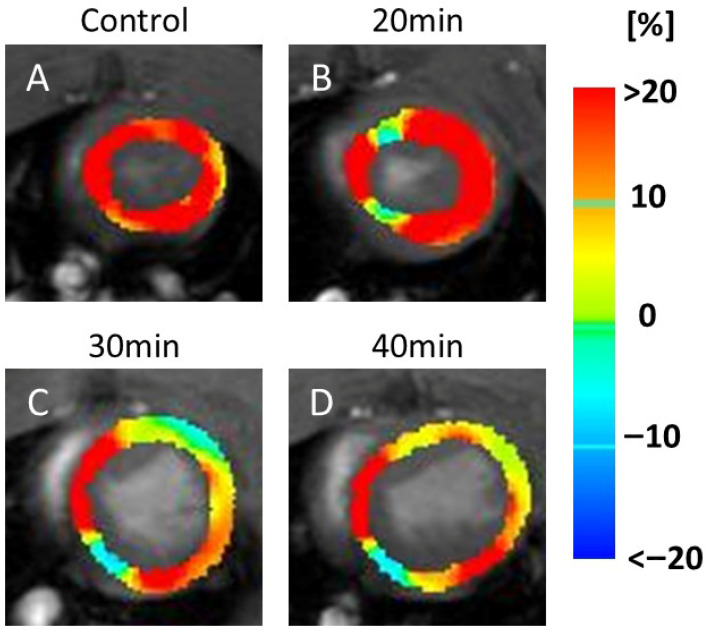
Color map of radial strain (RS). The image shows a series of MRI scans of the heart, labeled (**A**–**D**), representing the control and 20, 30, and 40 min post-infarction groups, respectively. Each scan is overlaid with a color map indicating strain values in the heart muscle: blue (<−20%), green (0%), and red (>20%). The scans illustrate changes in strain distribution over time, relevant for assessing cardiac function. Blue indicates motor decline. Compared with the control (**A**), a partial decrease in motion was observed at 20 min after infarction (**B**), while a more widespread decrease was seen at 30 min (**C**) and 40 min (**D**).

In the RS maps, representative images (Figure 4) demonstrated color changes that were consistently observed across all animals in each group. In the 20 min infarction group (Figure 4B), a partial reduction was evident compared to the control (Figure 4A). At 30 and 40 min (Figure 4C,D), yellow and light-blue areas extended throughout the myocardium, indicating reduced RS values in all animals in these groups.

**Figure 5:**
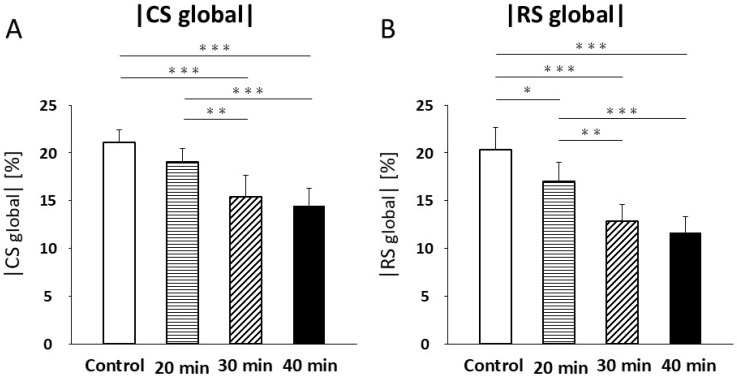
Circumferential strain (CS) (**A**) decreased at 30 and 40 min of infarction compared to controls and 20 min of infarction (*p* < 0.001). Radial strain (RS) (**B**) decreased at 20 min of infarction compared to controls (*p* < 0.05). RS (**B**) further decreased at 30 min and 40 min of infarction compared to controls (*p* < 0.001) and was lower at 30 min (*p* < 0.01) and 40 min (*p* < 0.001) than at 20 min of infarction. * *p* < 0.05; ** *p* < 0.01; *** *p* < 0.001.

CS values were not significantly different between the control and 20 min infarction groups (21.1 ± 1.3% vs. 19.1 ± 1.4%; Figure 5A, Table 2). A significant decrease in CS was observed in the 30 min infarction group (15.4 ± 2.2%) compared with both the control (*p* < 0.001) and the 20 min infarction group (*p* < 0.01; Figure 5A, Table 2). Similarly, CS was significantly lower in the 40 min infarction group (14.5 ± 1.8%, *p* < 0.001) compared with both control and 20 min infarction groups (Figure 5A, Table 2). No significant difference was found between the 30 min and 40 min groups (Figure 5A, Table 2). RS showed a significant reduction between the control and 20 min infarction groups (40.6 ± 4.7% vs. 34.0 ± 4.1%, *p* < 0.05; Figure 5B, Table 2). RS in the 30 min infarction group (25.6 ± 3.6%) was significantly lower than in the control (*p* < 0.001) and 20 min groups (*p* < 0.01, Figure 5B). RS was also significantly lower in the 40 min infarction group (23.4 ± 3.3%) compared to the control (*p* < 0.001) and 20 min infarction groups (*p* < 0.01; Figure 5B, Table 2). No significant difference in RS was observed between the 30 min and 40 min groups.

### 3.4. Longitudinal Strain

**Figure 6:**
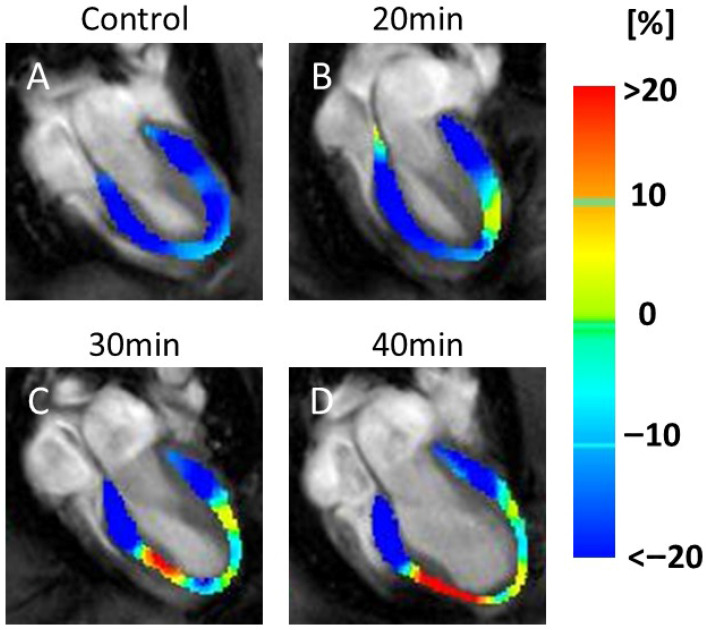
Color map of 4-chamber longitudinal strain (LS). MRI scans of the heart, labeled (**A**–**D**), represent the control, 20, 30, and 40 min post-infarction groups, respectively. Each scan was overlaid with a color map indicating strain values in the heart muscle. Blue represents strain values < −20%, green indicates 0%, and red indicates strain values > 20%. The scans illustrate changes in strain distribution over time. Red indicates motion decline. Motion weakness was observed at 30 min (**C**) and 40 min (**D**) after infarction compared to the control (**A**) and 20 min (**B**).

Figure 6 shows color maps of the four-chamber LS (Figure 6A–D). In the four-chamber LS maps, increased red areas, indicating decreased LS, were seen at 30 min (Figure 6C) and 40 min (Figure 6D) after infarction.

**Figure 7:**
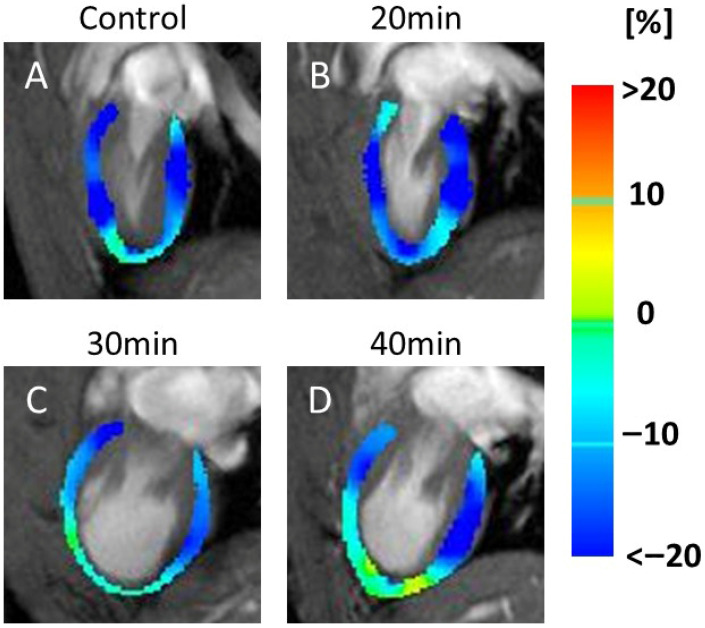
Color map of 2-chamber longitudinal strain (LS). MRI scans of the heart, labeled (**A**–**D**), represent the control, 20, 30, and 40 min post-infarction groups, respectively. Each scan was overlaid with a color map indicating strain values in the heart muscle. Blue represents strain values < −20%, green indicates 0%, and red indicates strain values > 20%. The scans illustrate changes in strain distribution over time. Yellow indicates motion decline. Motion weakness was seen at 30 min (**C**) and 40 min (**D**) after infarction compared to the control (**A**) and 20 min (**B**).

Figure 7 shows the color maps of the two-chamber LS (Figure 7A–D). These images displayed an increase in yellow regions, indicating decreased motion, at 30 min (Figure 7C) and 40 min (Figure 7D).

**Figure 8:**
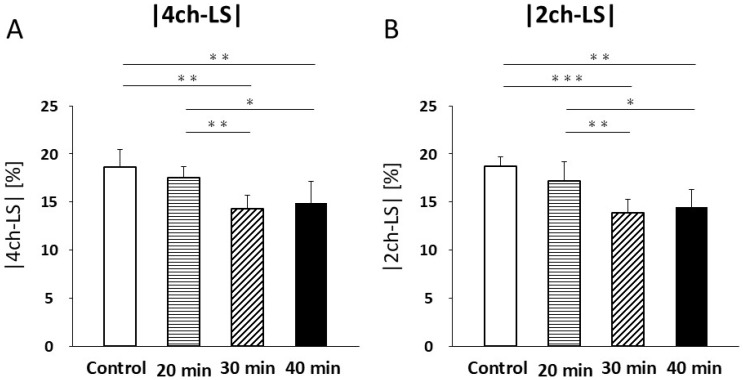
Four-chamber longitudinal strain (LS) (**A**) decreased at 30 min and 40 min of infarction compared to control (*p* < 0.01) and 20 min of infarction (30 min, *p* < 0.01; 40 min, *p* < 0.05). An increasing trend in LS was noted at 40 min compared to that at 30 min. Two-chamber LS (**B**) decreased at 30 and 40 min of infarction compared to control (30 min, *p* < 0.001; 40 min, *p* < 0.01) and 20 min (30 min, *p* < 0.01; 40 min, *p* < 0.05). An increasing trend in LS was also observed at 40 min compared to that at 30 min. GLS values were re-plotted as negative values, consistent with convention. * *p* < 0.05; ** *p* < 0.01; *** *p* < 0.001.

In four-chamber LS, no significant difference was found between the control and 20 min infarction groups (18.6 ± 1.8% vs. 17.6 ± 1.1%; Figure 8A, Table 3). Significant decreases were observed in the 30 min infarction group (14.3 ± 1.4%, *p* < 0.01) and the 40 min group (14.9 ± 2.3%) compared with both the control (*p* < 0.01, Figure 8A, Table 3) and 20 min groups (*p* < 0.05, Figure 8A, Table 3). No significant differences were observed between the 30 min and 40 min groups.

In the two-chamber LS, no significant differences were noted between the control and 20 min groups (18.8 ± 1.0% vs. 17.2 ± 2.0%; Figure 8B, Table 3). However, the 30 min infarction group (13.9 ± 1.4%) was significantly lower than that in the control (*p* < 0.001) and 20 min groups (*p* < 0.01, Figure 8B, Table 3). Similarly, the 40 min group (14.5 ± 1.8%) was significantly reduced compared to the control (*p* < 0.01, Figure 8B, Table 3) and 20 min groups (*p* < 0.05; Figure 8B). No significant differences were observed between the 30 min and 40 min groups.

### 3.5. Tissue Staining

**Figure 9:**
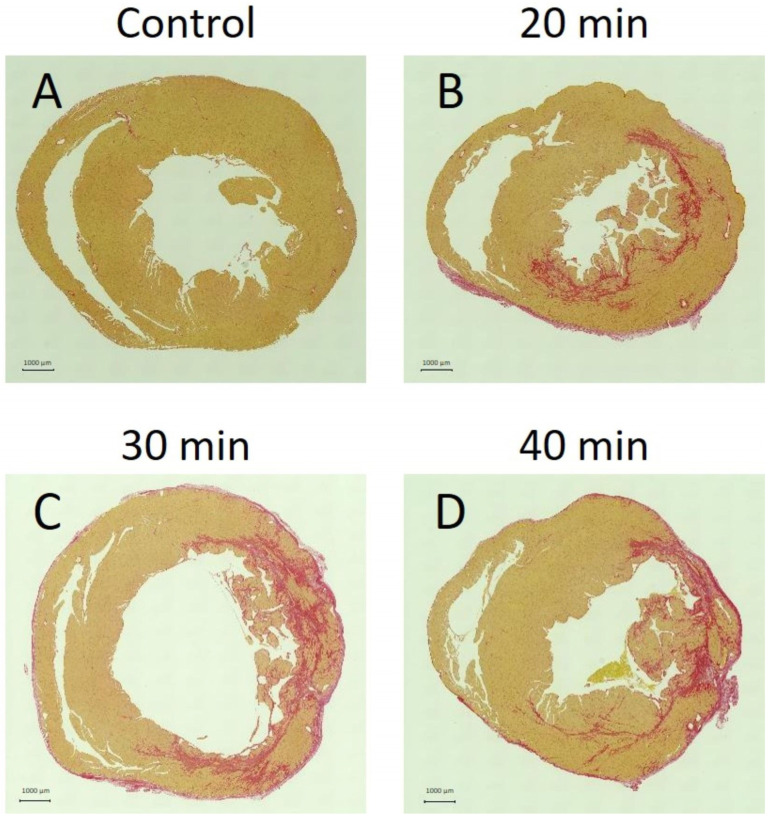
Panels A–D show tissue sections stained with Sirius Red, where red-stained areas indicate fibrosis. No fibrosis was observed in controls (**A**). Fibrosis appeared at 20 min (**B**), 30 min (**C**), and 40 min (**D**) after infarction. At 20 min, fibrosis was confined to the endocardium, whereas at 30 and 40 min, it extended beyond the endocardium into the outer myocardial layers.

Fibrosis was absent in the control group (Figure 9A) but present in the 20 min (Figure 9B), 30 min (Figure 9C), and 40 min (Figure 9D) infarction groups. In the 20 min infarction group, fibrosis was confined to the endocardium, whereas in the 30 min and 40 min groups, it extended to the epicardium.

**Figure 10:**
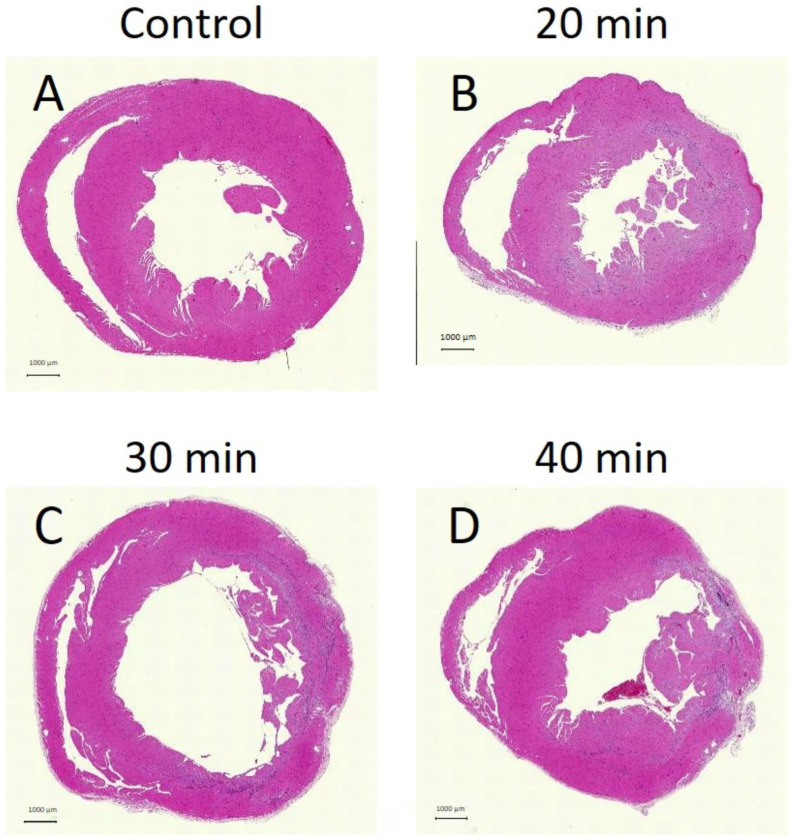
Hematoxylin and eosin (HE)-stained tissue sections. Lighter-stained areas were visible at 20, 30, and 40 min after infarction (**B**–**D**), compared to the control (**A**). Necrosis was observed in the endocardium at 20 min, while by 40 min, it extended to the epicardium.

HE staining appeared weaker in the 20 min (Figure 10B), 30 min (Figure 10C), and 40 min (Figure 10D) groups compared to controls (Figure 10A). Areas of weaker HE staining corresponded to regions of fibrosis on Sirius Red staining, indicating necrosis.

## 4. Discussion

This study employed a rat model of coronary artery reperfusion subjected to infarction durations of 20, 30, and 40 min and utilized cardiac cine-MRI with preclinical 7T-MRI to evaluate both cardiac function and strain values. MRI-derived parameters of cardiac function showed no significant differences between the control and 20 min infarction groups; however, marked differences emerged at 30 and 40 min. Notably, only RS demonstrated a significant reduction at 20 min compared with controls. Moreover, Sirius Red staining revealed that fibrosis was confined to the endocardium at 20 min, suggesting mild myocardial damage, which correlated with the reduction in RS, as no substantial changes were observed in other parameters at this time point. Thus, RS may serve as an early marker of short-duration myocardial reperfusion injury. Because imaging occurred at 1 week, the present results represent early subacute rather than purely acute changes. Early imaging (24–48 h) may yield different relative sensitivities of RS and GLS due to myocardial stunning, edema, and evolving necrosis. These temporal differences should be considered when comparing our findings with acute-phase studies or clinical imaging performed shortly after reperfusion.

MRI strain analysis is a valuable technique for assessing remodeling during myocardial infarction [15,16,17,18]. In addition to RS, other myocardial strain parameters did not change significantly in the 20 min infarction group compared to those in the 30 and 40 min groups. The exclusively early reduction in RS suggests its potential utility as an early marker of myocardial infarction in humans. Emerging data indicate that remote myocardium exhibits subtle strain and mapping abnormalities proportional to the global injury burden [19]. Although our primary endpoints were global, future work will incorporate regional strain (infarct/core, peri-infarct, remote) and compare them with histologic fibrosis to quantify the contribution of remote remodeling. During remodeling following infarction, healthy cardiomyocytes lengthen and undergo progressive hypertrophy to maintain normal stroke volume despite fewer functioning myocardial segments and increased workload. This elongation may alter myocardial strain [20,21]. Notably, cardiac cine-MRI using preclinical 7T-MRI has emerged as a powerful tool for analyzing cardiac function and myocardial deformation in infarction–reperfusion models.

We did not perform predefined remote-vs.-infarct analyses; future studies will employ segmental strain and transmural fibrosis scoring to test correlations with global and regional strain. Although no significant differences were observed between the control and 20 min infarction groups in ESV, EDV, or EF, significant differences were found between the control and 30 min groups, as well as between the 20 and 30 min groups. Similarly, significant differences were noted between the control and the 40 min infarction group. These findings indicate that infarctions lasting ≥30 min significantly impair cardiac function. Nonetheless, Sirius Red staining revealed myocardial damage as early as 20 min, underscoring the limitations of relying solely on ESV, EDV, and EF for assessing short-duration infarctions. While these parameters are well-established as indicators of cardiac function and remodeling, evidence shows that EF alone is insufficient [22,23]. Over the last decade, studies have demonstrated that global LS (GLS) is more sensitive to left-ventricular (LV) dysfunction compared with LVEF and provides superior prognostic value [23,24]. Our results support this evidence, showing that GLS is more sensitive to LV dysfunction than LVEF.

In addition, RS showed a significant decline at 30 min, reflecting a substantial reduction in myocardial deformation coinciding with impaired cardiac function. Previous studies have similarly reported reduced EF 30 min after infarction [11], consistent with our findings. Sirius Red staining illustrated initial fibrotic changes confined to the endocardium at 20 min, which extended to the epicardium beyond 30 min. Myocardial infarction typically begins in the endocardium and progresses outward [25]. As fibrosis and necrosis advance from non-penetrating to penetrating, myocardial viability declines [26]. In this study, 20 min of infarction produced mild damage, with greater severity as duration increased beyond 30 min. Among ESV, EDV, EF, RS, CS, four-chamber LS, and two-chamber LS, significant differences were observed between the control and 30 min groups, as well as between the 20 and 30 min groups, paralleling histological findings. These results indicate that infarctions lasting >30 min substantially impair cardiac function and exacerbate myocardial deformation. The earlier decline in RS likely reflects the vulnerability of subendocardial layers that contribute disproportionately to radial thickening via circumferential fiber shortening and sheet reorientation, whereas GLS can be transiently buffered by compensatory longitudinal shortening in remote myocardium. This fiber-architecture dependence and deformation physics have been detailed in recent methodological reviews [27], providing a physiological rationale for RS as an earlier indicator of injury.

In patients with reperfused MI, we propose a pragmatic CMR strategy that pairs feature-tracking GLS (and RS where feasible) with LGE and T1/T2 mapping to capture both deformation and tissue characterization. Given our preclinical signal that RS declines earlier, early post-PCI CMR could test RS as an early-warning readout complementary to GLS and LGE. While absolute strain values differ across field strengths and vendors, the directionality (RS earlier than GLS) is clinically testable and may refine risk stratification beyond LVEF.

In summary, most parameters showed no significant differences between the control and 20 min infarction groups, with notable changes emerging only at 30 min. However, RS alone demonstrated a significant decline at 20 min. Sirius Red staining revealed localized fibrosis confined to the endocardium at this time, suggesting mild myocardial damage consistent with the RS decrease, given the absence of changes in other parameters. Sirius Red staining serves as an indicator of fibrosis following myocardial infarction, reflecting the interval between infarction and reperfusion [14,28,29]. Consequently, RS appears capable of detecting myocardial damage earlier than other parameters. Prior studies have indicated that RS changes occur earlier and are more pronounced than LS changes, suggesting RS as a potential early detection marker for myocardial infarction [30], which aligns with the current findings. Together, these results delineate a duration-dependent hierarchy of impairment and nominate RS as a candidate early marker for translation.

No significant difference in LS was observed between the 30 and 40 min groups. In a previous study using a model of complete coronary artery occlusion, LS was reported to increase within the ischemic myocardium [15]. This is thought to occur because diminished circumferential and radial motion in the ischemic myocardium is compensated for by enhanced longitudinal motion in the non-ischemic myocardium. This compensatory mechanism is consistent with our results, supporting a tendency for LS to rise in severely ischemic myocardium.

Our findings are consistent with those of previous reports demonstrating a critical threshold of approximately 30 min of coronary occlusion for the onset of significant left-ventricular dysfunction. Arias et al. [11] similarly observed a decline in EF in rats after 30 min of ischemia, consistent with our data. Moreover, the early sensitivity of RS in our study parallels prior work showing that radial strain alterations occur earlier than longitudinal changes in experimental models [30]. Clinically, our observation that GLS is more sensitive than EF supports existing evidence that GLS provides incremental prognostic value over LVEF in ischemic heart disease [8]. In addition, the progression of fibrosis from the endocardium toward the epicardium in our histological analysis corresponds well to the “wavefront phenomenon” of myocardial ischemic injury described by Reimer and Jennings [25]. Taken together, these comparisons underscore that our preclinical 7T-MRI findings not only replicate known patterns of ischemia–reperfusion injury but also highlight the translational potential of strain analysis as a sensitive tool for detecting early myocardial dysfunction.

This study had some limitations worth noting. Cine-MRI was used to evaluate cardiac function and myocardial deformation through strain analysis. However, the actual extent of myocardial damage remains unclear. Future studies should incorporate additional histological methods to correlate myocardial damage with cine-MRI findings. Furthermore, late gadolinium enhancement, widely used clinically to visualize injured myocardium, was not employed in this study because T1 mapping is not feasible in rodents using 7T-MRI. Future investigations should, therefore, consider incorporating late gadolinium enhancement alongside strain analysis. Larger sample sizes are also needed to validate these results. Although we calculated global strain by assessing the entire myocardium, evaluating individual slices or regional strain could provide additional insights. Moreover, rodent models differ from humans in cardiac and coronary anatomy as well as injury patterns. Therefore, similar studies in large animal models of myocardial infarction, such as pigs, are required. Finally, our experiments used a coronary artery reperfusion model involving occlusion of the left main coronary artery trunk. Given the extensive regions supplied by the left main coronary artery, occlusion and reperfusion procedures applied to vessels at lower levels, such as the left anterior descending coronary artery and left circumflex artery, could reveal more localized effects. Detailed investigations at the regional and slice-specific levels are warranted in future research.

In this preclinical 7 T reperfusion model, graded increases in ischemia duration were associated with stepwise impairments in global function and strain at 1 week. Notably, RS exhibited an earlier reduction than GLS/CS/LVEF, consistent with subendocardial vulnerability. While these findings generate a testable translational hypothesis—that RS may detect injury earlier than GLS and LVEF—confirmatory studies with larger samples, regional analyses, and clinical CMR are warranted before inferring patient-level thresholds.

## 5. Conclusions

Cardiac function and myocardial deformation worsened with increasing reperfusion time, with significant deterioration observed at 30 min after infarction. Cardiac cine-MRI using 7T-MRI proved to be a valuable tool for assessing cardiac function and myocardial deformation in a myocardial infarction–reperfusion model.

## Figures and Tables

**Table 1:** Cardiac function.

	Control	20 min	30 min	40 min
ESV [mL]	0.13 ± 0.02	0.14 ± 0.01	0.26 ± 0.08 ***###	0.26 ± 0.06 ***###
EDV [mL]	0.33 ± 0.02	0.34 ± 0.02	0.45 ± 0.08 **##	0.44 ± 0.78 *#
EF [%]	62.09 ± 3.79	59.18 ± 2.18	43.99 ± 7.73 ***###	42.73 ± 5.36 ***###

vs.control/*: *p* < 0.05, **: *p* < 0.01, ***: *p* < 0.001. vs.20 min/#: *p* < 0.05, ##: *p* < 0.01, ###: *p* < 0.001.

**Table 2:** Circumferential strain (CS) and radial strain (RS).

	Control	20 min	30 min	40 min
|CS global| [%]	21.07 ± 1.32	19.09 ± 1.37	15.43 ± 2.21 ***###	14.48 ± 1.83 ***###
|RS global| [%]	40.62 ± 4.68	33.97 ± 4.09 *	25.67 ± 3.56 ***##	23.4 ± 3.31 ***###

vs.control/*: *p* < 0.05, ***: *p* < 0.001. vs.20 min/##: *p* < 0.01, ###: *p* < 0.001.

**Table 3:** Four-chamber longitudinal strain (LS) and two-chamber LS.

	Control	20 min	30 min	40 min
|lS_4ch| [%]	18.60 ± 1.83	17.55 ± 1.10	14.27 ± 1.44 **##	14.87 ± 2.27 **#
|lS_2ch| [%]	18.75 ± 0.98	17.16 ± 2.02	13.85 ± 1.41 ***##	14.47 ± 1.79 **#

vs.control/**: *p* < 0.01, ***: *p* < 0.001. vs.20 min/#: *p* < 0.05, ##: *p* < 0.01.

## Data Availability

The datasets generated and/or analyzed during the current study are available from the corresponding author upon reasonable request.

## Abbreviations

The following abbreviations are used in this manuscript:

CI	Confidence interval
GLS	Global longitudinal strain
HE	Hematoxylin and eosin
LS	Longitudinal strain
LVEF	Left-ventricular ejection fraction
MRI	Magnetic resonance imaging
RS	Radial strain
CS	Circumferential strain
